# Supplementary material for: In-orbit operation of an atomic clock based on laser-cooled 87Rb atoms
Source: Nat Commun. 2018 Jul 24;9:2760. doi: 10.1038/s41467-018-05219-z (PMC6057979; doi:10.1038/s41467-018-05219-z)
Supplement: Supplementary file 1 — Supplementary Figures [file 41467_2018_5219_MOESM1_ESM.pdf]

Supplementary Figures

In-orbit operation of an atomic clock based on laser-cooled ^{87}Rb atoms

Liu et al.

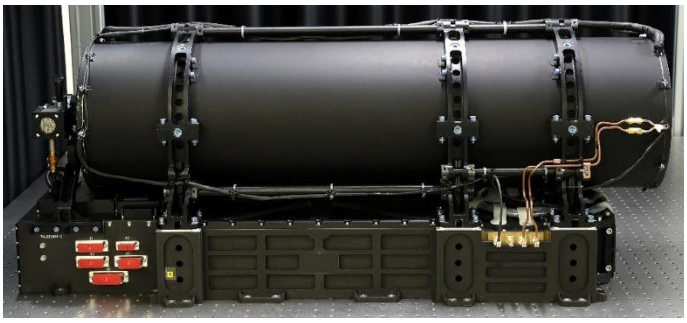

Supplementary Figure 1 **Photograph of the flight model of the CACES's setup.** The upper part of the setup is the physics package including an ultra-high vacuum tube inside the three-layer shield. The bottom part consists of control electronics, optical bench, and microwave source (from left to right).

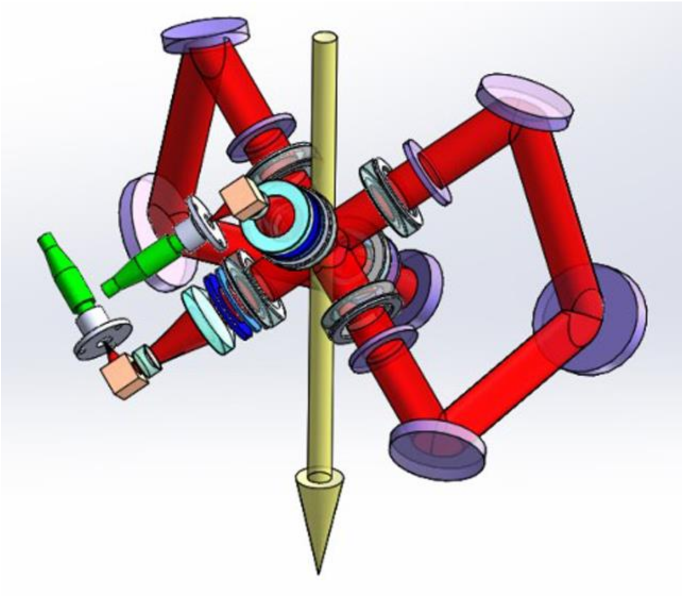

(a)

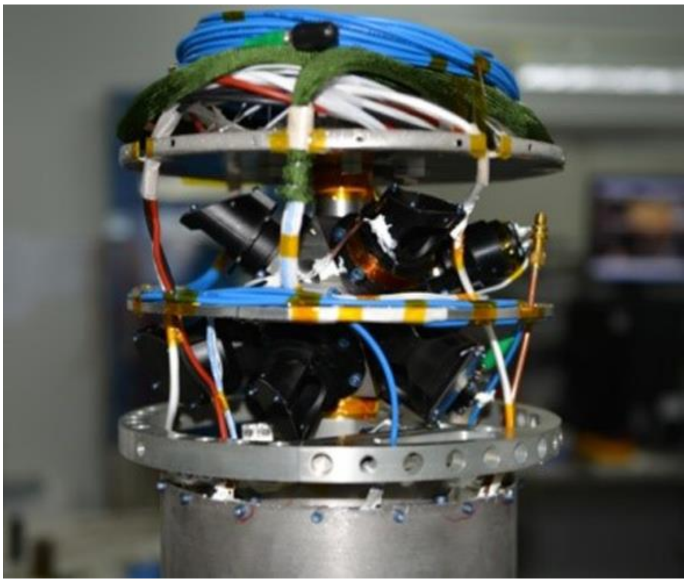

(b)

Supplementary Figure 2 **The configuration of the MOT setup**. Two laser beams are folded to form three pair orthogonal laser beams as shown in (a). The photograph of the setup is as shown in (b).

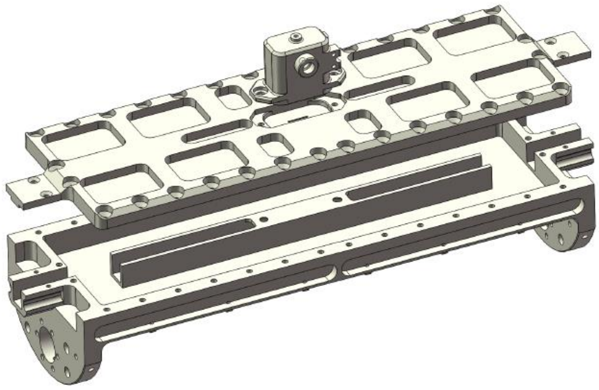

Supplementary Figure 3 **Explosive view of the interrogation cavity.** The interrogation cavity consists of the coupled waveguide, the cover and the main body.

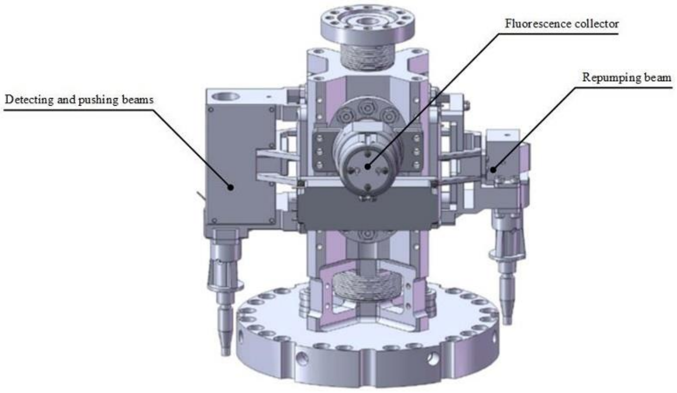

Supplementary Figure 4 **Configuration of the detection system.** The setup consists of three laser beams, including a detecting beam, a pushing beam and a repumping beam. The fluorescence collector includes two photodiodes. This configuration ensures us to detect the cold atoms in both hyperfine states simultaneously.

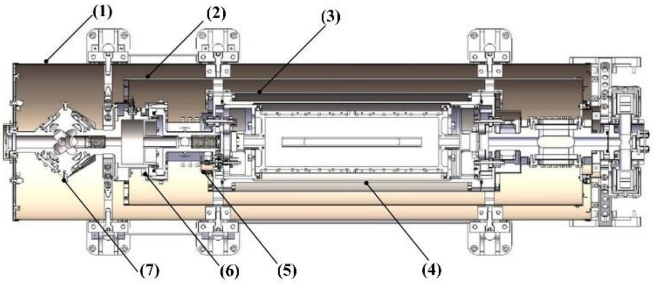

Supplementary Figure 5 **Magnetic architecture of the CAC.** (1) outer shield; (2) middle shield; (3) inner shield; (4) C-field coil for the interrogation zone; (5) compensation coil for the interrogation zone; (6) C-field coil for the state selection zone; (7) compensation coil for the MOT.

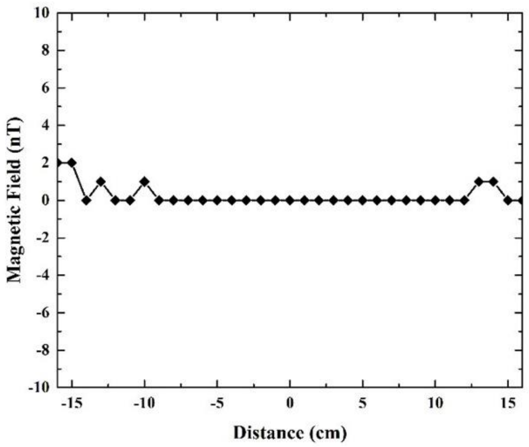

Supplementary Figure 6 **Axial variation of the magnetic field inside the shield under static geomagnetic condition.** The zero position of distance is defined at the axial center of the interrogation cavity.

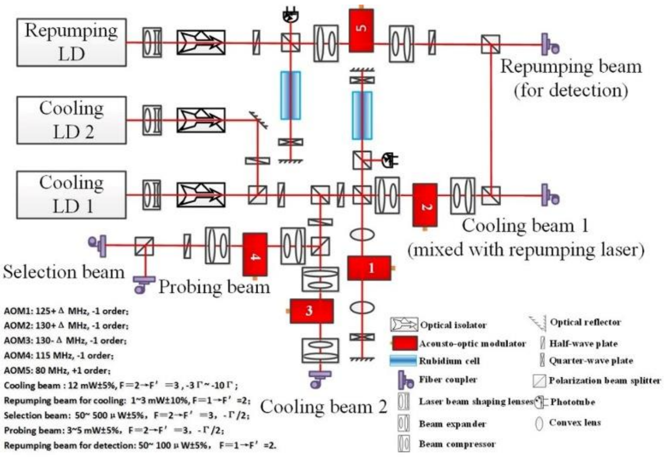

Supplementary Figure 7 **Architecture of the optical bench.** Cooling LD 2 is the backup of cooling LD 1. All the lasers are frequency locked to the saturated absorption signal. The laser beam from cooling LD is divided into cooling beams, selection beam and probing beam. Repumping LD provides the repumping beams for cooling and detection. Five AOMs are used to adjust the frequency and power of each beam.

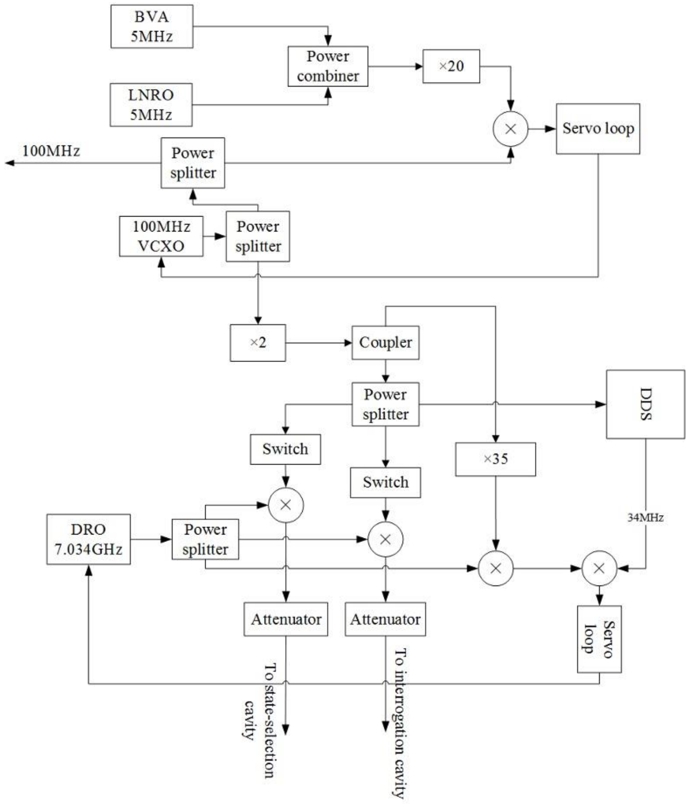

Supplementary Figure 8 **Schematic of the microwave source.** A low noise reference oscillator (LNRO) is the backup of the oscillator of BVA8607. The 5 MHz reference frequency is multiplied and synthesized to rubidium clock transition frequency of 6.8 GHz.

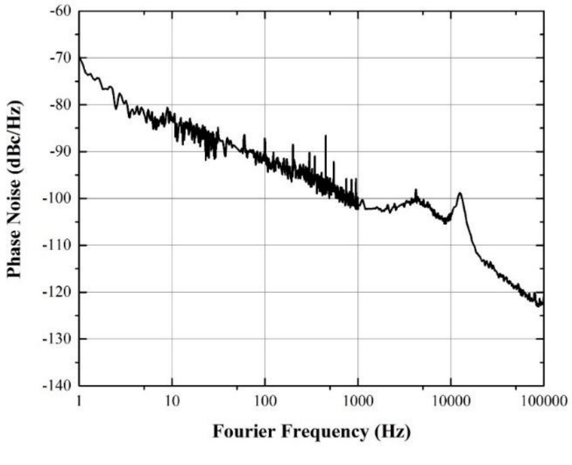

Supplementary Figure 9 **The measured phase noise of the microwave source at 6.8 GHz.** This result is obtained by pair measurement.

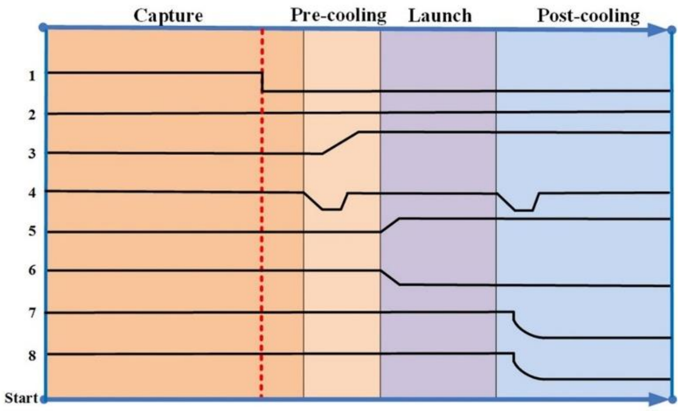

Supplementary Figure 10 **Timing sequence of the laser trapping and cooling.** (1) current in the MOT coil; (2) laser power for frequency stabilization; (3) cooling laser frequency regulated by AOM 1; (4) cooling laser frequency regulated by the laser current; (5) frequency of cooling beam 1 regulated by AOM 2; (6) frequency of cooling beam 2 regulated by AOM 3; (7) power of cooling beam 1 regulated by AOM 2; (8) power of cooling beam 2 regulated by AOM 3.
